# Temporal Trends of Intravenous Thrombolysis Utilization in Acute Ischemic Stroke in a Prospective Cohort From 1998 to 2019: Modeling Based on Joinpoint Regression

**DOI:** 10.3389/fneur.2022.851498

**Published:** 2022-04-08

**Authors:** Verónica V. Olavarría, Lorena Hoffmeister, Carolina Vidal, Alejandro M. Brunser, Arnold Hoppe, Pablo M. Lavados

**Affiliations:** ^1^Unidad de Neurología Vascular, Servicio de Neurología, Departamento de Neurología y Psiquiatría, Clínica Alemana de Santiago, Facultad de Medicina Clínica Alemana, Universidad del Desarrollo, Santiago, Chile; ^2^Escuela de Salud Pública, Facultad de Medicina, Universidad Mayor, Santiago, Chile; ^3^Unidad de Investigación y Ensayos Clínicos, Departamento de Desarrollo Académico e Investigación, Clínica Alemana de Santiago, Facultad de Medicina Clínica Alemana Santiago, Universidad del Desarrollo, Santiago, Chile

**Keywords:** intravenous thrombolysis, ischemic stroke, door-to-needle time, performance measures, Joinpoint regression, behavioral change wheel, implementation

## Abstract

**Introduction:**

The frequency of intravenous thrombolysis (IVT) in acute ischemic stroke (AIS) is lower than it should be in several regions of the world. It is unclear what interventions can produce significant improvements in IVT utilization. We aimed to investigate the temporal trends in IVT in AIS and identify changes in time that could be associated with specific interventions.

**Methods:**

We included patients with AIS who were admitted from January 1998 to December 2019 in our institution. To analyze trends in utilization and time points in which they changed, we performed a Joinpoint regression analysis. Interventions were assigned to a specific category according to the Behavior Change Wheel framework intervention function criteria.

**Results:**

A total of 3,361 patients with AIS were admitted, among which 538 (16%) received IVT. There were 245 (45.5%) women, and the mean age and median National Institutes of Health Stroke Scale (NIHSS) scores were 68.5 (17.2) years and 8 (interquartile range, 4–15), respectively. Thrombolysis use significantly increased by an average annual 7.6% (95% CI, 5.1–10.2), with one Joinpoint in 2007. The annual percent changes were.45% from 1998 to 2007 and 9.57% from 2007 to 2019, concurring with the stroke code organization, the definition of door-to-needle times as an institutional performance measure quality indicator, and the extension of the therapeutic window.

**Conclusions:**

The IVT rates consistently increased due to a continuous process of protocol changes and multiple interventions. The implementation of a complex multidisciplinary intervention such as the stroke code, as well as the definition of a hospital quality control metric, were associated with a significant change in this trend.

## Introduction

Intravenous thrombolysis (IVT) for patients with acute ischemic stroke (AIS) is efficacious and safe (1, 2).

However, the frequency of IVT in AIS is still lower than it should be to say that it has a significant population effect in many countries and regions of the world (3–6). Several barriers and reasons have been described for this, which have usually been classified in two. First (a) is during pre-admission, including patient-related (poor symptom recognition, late presentation, and narrow time window for IVT) and paramedic-related (time delays and transport to not-ready hospitals) barriers. Second (b) is during post-admission, including hospital-related barriers (suboptimal triage systems, inefficient stroke care pathways, lack of infrastructure or expertise), physician choices (experience and comfort with uncertainties), and economic/political environments (high costs, quality control, or accreditation) (7–10).

Intravenous thrombolysis is a complex multilevel, multidisciplinary decision-making process. Since its approval, its utilization has been timidly increasing, being unclear what interventions are the ones likely to produce the most significant increase in its use. Four recent systematic reviews and meta-analyses have investigated this question. Paul et al. using the Behavior Change Wheel (BCW) framework found that access to teaching hospitals and hospitals with large stroke and IVT volumes is associated with increased thrombolysis rates (11, 12). Using the same framework, Hasnain focused the analysis on the following five intervention strategies: education, persuasion, training, environmental restructuring, and enablement. All approaches were found to increase thrombolysis uptake at a similar rate, and the subgroup analysis suggested that persuasion, environmental restructuring, and enablement may be particularly effective (13). Huang et al. found that optimization of workflow with organizational improvements, including the centralization of stroke care and prenotification or telemedicine, contributed the most to reducing pre- and in-hospital delays (14). McDermott et al. found that interventions targeted at emergency medical services (EMS), telemedicine, and public education were associated with a trend toward an increase in IVT (15).

We aimed to investigate significant changes in the temporal trends of IVT in AIS in our institution and identify interventions that could be associated, according to the BCW framework. The secondary outcomes were usual time metrics and compliance with performance measures.

## Materials and Methods

In this cohort study, we analyzed data from our prospective stroke registry (Registro de Enfermedades Cerebrovasculares Clínica Alemana [RECCA]), which included all adult patients (aged ≥ 18 years) with acute stroke admitted since 1997. These patients consented to participate in the current study. Our institution is a teaching nonprofit tertiary private hospital in Santiago, Chile.

We selected patients with AIS admitted from January 1998 to December 2019. We excluded patients with transient ischemic attacks, which were defined according to time and imaging results, and those with spinal cord or retinal infarctions and stroke mimics (16). All patients with suspected stroke presenting to the emergency department were assessed by an on-call neurologist. All variables were prospectively collected during patient hospitalization and transferred to an electronic database REDCap electronic data capture tools hosted at Clínica Alemana. REDCap (Research Electronic Data Capture, Vanderbilt University, Nasshville, TN, USA). All admitted stroke patients were identified and recruited by physician investigators who screen stroke admissions daily. The neurology staff met weekly for a case audit with the focus on protocol deviations. Protocols were reviewed annually or biannually depending on emerging new evidence.

Ischemic stroke is defined as an episode of neurological dysfunction caused by focal cerebral infarction according to the current standard definitions (16). Hypertension, diabetes mellitus, hyperlipidemia, cardiopathies, and atrial fibrillation are defined as present in patients with the previous clinical diagnosis or those under treatment for each specific risk factor.

The stroke program was organized formally in late 1997 and included the first protocol for IVT with recombinant tissue plasminogen (r-TPA), which was based on the National Institute of Neurological Disorders and Stroke trial (17). Since then, significant organization and protocol changes have taken place. These were based on newly published evidence or guidelines and the consensus of the vascular neurology staff. Each intervention is described in detail and assigned to specific categories of interventions as suggested by the BCW framework intervention function criteria in Supplementary Tables 1, 2 (12).

In this exploratory research, our primary objective was to identify significant changes in trends of the IVT frequency of use. The secondary objectives were to identify significant changes in onset-to-door (OTD) times (pre-hospital delay), door-to-needle (DTN) times (in-hospital delay), and onset-to-needle (OTN) times (total delay). We also aimed to identify significant changes in the frequency of patients arriving within 120 min of OTD, as well as in the frequency of thrombolysis performed within 60 min of DTN times and within 180 min of OTN times (18).

### Statistical Analyses

The total thrombolysis cohort was described with means and *SD*s or medians and interquartile ranges (IQRs) for continuous or ordinal data, respectively. Frequencies were presented with percentages (%) and 95% CIs.

To analyze trends in IVT use and identify the time point(s) in which the trends significantly changed, we performed a Joinpoint regression analysis. The method calculates the annual percentage change (APC) in rates between trend-change points, and also estimates the average annual percentage change (AAPC) in the whole period studied. When there are no changes in trend (i.e., no Joinpoint), APC is constant, so it equals the AAPC. Otherwise, the whole period is segmented by the points with a trend change. We chose a data-driven Bayesian information criterion (BIC) method, namely the weighted BIC (19).

The primary outcome was analyzed by Joinpoint in the total cohort of patients treated with thrombolysis as frequency per year. Joinpoint for symptom OTD, DTN, and symptom OTN times was analyzed according to the mean minutes per year. Compliance with performance measures was analyzed as annual Joinpoint in the trends of the percentage of patients being admitted within 120 min of symptom onset, being treated with thrombolysis within 60 min of presentation to the emergency department per year, and being treated with thrombolysis within 180 of symptom onset. Missing data were excluded from the analyses.

All statistical analyses were performed with the Joinpoint trend analysis Program Version 4.9.0.0 – March 25, 2021. National Cancer Institute, USA (19). An alpha error of <0.05 was considered significant. The article is reported according to the STROBE guidelines (16).

The scientific Ethics Committee of Universidad del Desarrollo and the Institutional Review Board of Clínica Alemana de Santiago approved the study registry protocol and written informed consent was obtained in every patient as local regulatory law requests.

## Results

From 1998 to 2019, 3,361 patients with AIS were admitted, among which 538 (16%) were treated with IVT. There were 245 (45.5%) women, the mean age of the treated patients was 68.5 (17.2) years, and the median National Institutes of Health Stroke Scale (NIHSS) score was 8 (IQR, 4–15). Table 1 describes their baseline demographic and clinical characteristics.

**Table 1:** Baseline characteristics of the whole cohort of patients treated with thrombolysis.

**Variable**	**Total *N* = 538**
Age, mean years (SD*)	68.5 (17.2)
Age > 80, (%)	155 (28.7)
Female, (%)	245 (45.5)
Hypertension, (%)	332 (61.7)
Diabetes mellitus, (%)	70 (13.0)
Dyslipidemia, (%)	177 (33.3)
Prior stroke, (%)	87 (16.2)
Any cardiopathy, (%)	192 (35.7)
Known atrial fibrillation, (%)	86 (16)
Current smoker, (%)	153 (28.4)
NIHSS^†^, median (IQR^‡^)	8 (4–15)
NIHSS 0–5, (%)	199 (37.3)
Systolic blood pressure, mean mmHg (SD)	152.6 (26.4)
Diastolic blood pressure, mean mmHg (SD)	84.6 (16.2)
Glucose, mean mg/dL (SD)	122.5 (43.3)
Symptom onset to door time, mean min (SD)	94.8 (97.3)
Symptom onset to door time, <120 min (%)	393 (73.0)
Door to needle time, mean min (SD)	56.4 (37.7)
Door to needle time, <60 min (%)	290 (53.9)
Symptom onset to needle, mean min (SD)	151.0 (98.2)
Symptom onset to needle, <180 min (%)	377 (70.1)

*SD, Standard deviation.

*NIHSS, National Institute of Health Stroke Scale.*

*^‡^IQR, Inter quartile range*.

**Figure 1:**
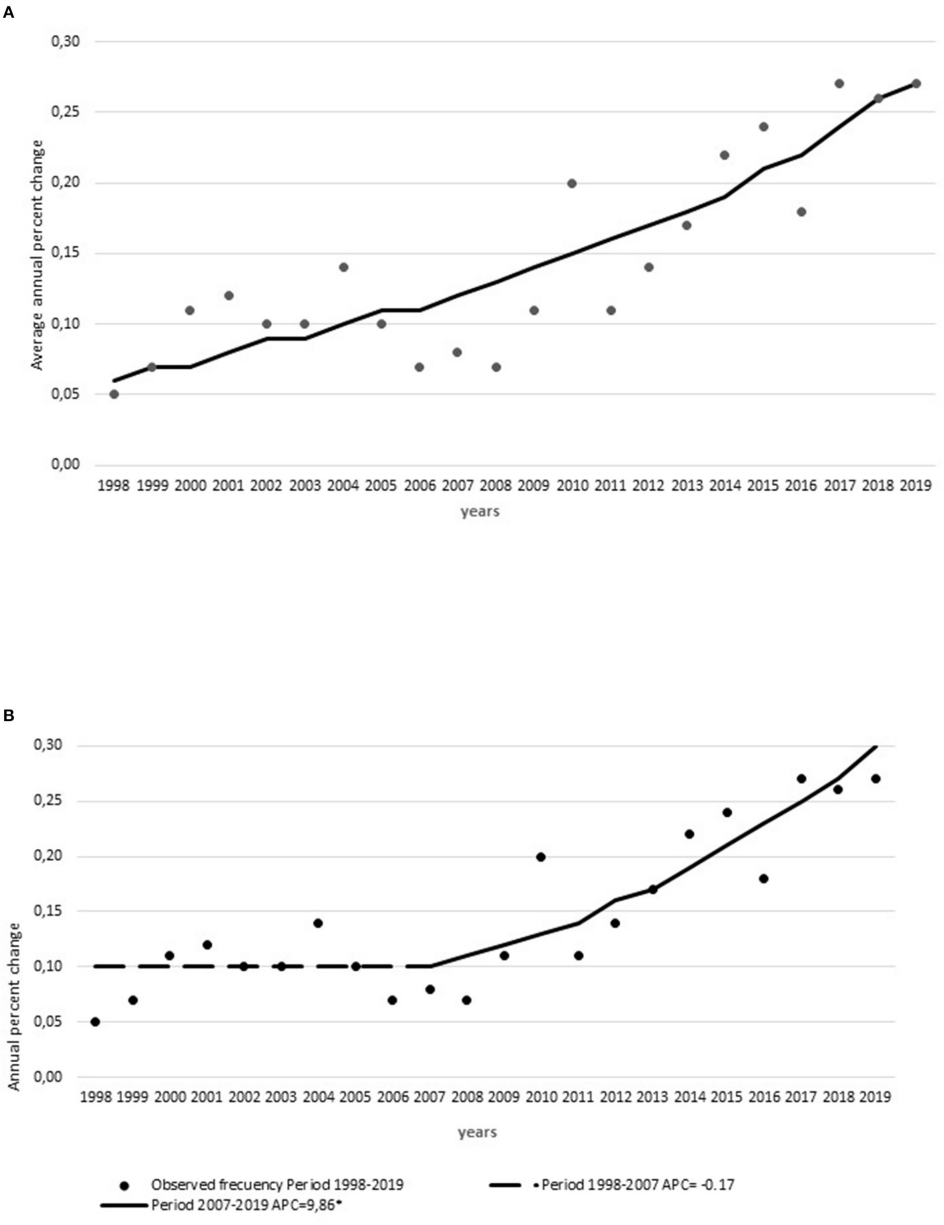
Thrombolysis utilization rates. **(A)** Average annual percent change of thrombolysis utilization rate and **(B)** annual percent change of thrombolysis utilization rate from 1998 to 2019 with Joinpoint organizational or protocol modifications from 1997 to 2019. *Indicates were the annual percent change (APC) is significantly different from zero at the alpha = 0.05 level.

The frequency of thrombolysis use significantly increased with an AAPC for thrombolysis utilization of 7.6% (95% CI, 5.1–10.2) from 1998 to 2019. The regression model for IVT utilization showed that there was one Joinpoint in 2007. The APCs were 0.45% from 1998 to 2007 and 9.57% from 2007 to 2019 (Figure 1). This coincided with (a) the stroke code organization, (b) the extension of the therapeutic window, and (c) the definition of DTN time of <60 min as an institutional quality control measure (Supplementary Table 2).

**Figure 2:**
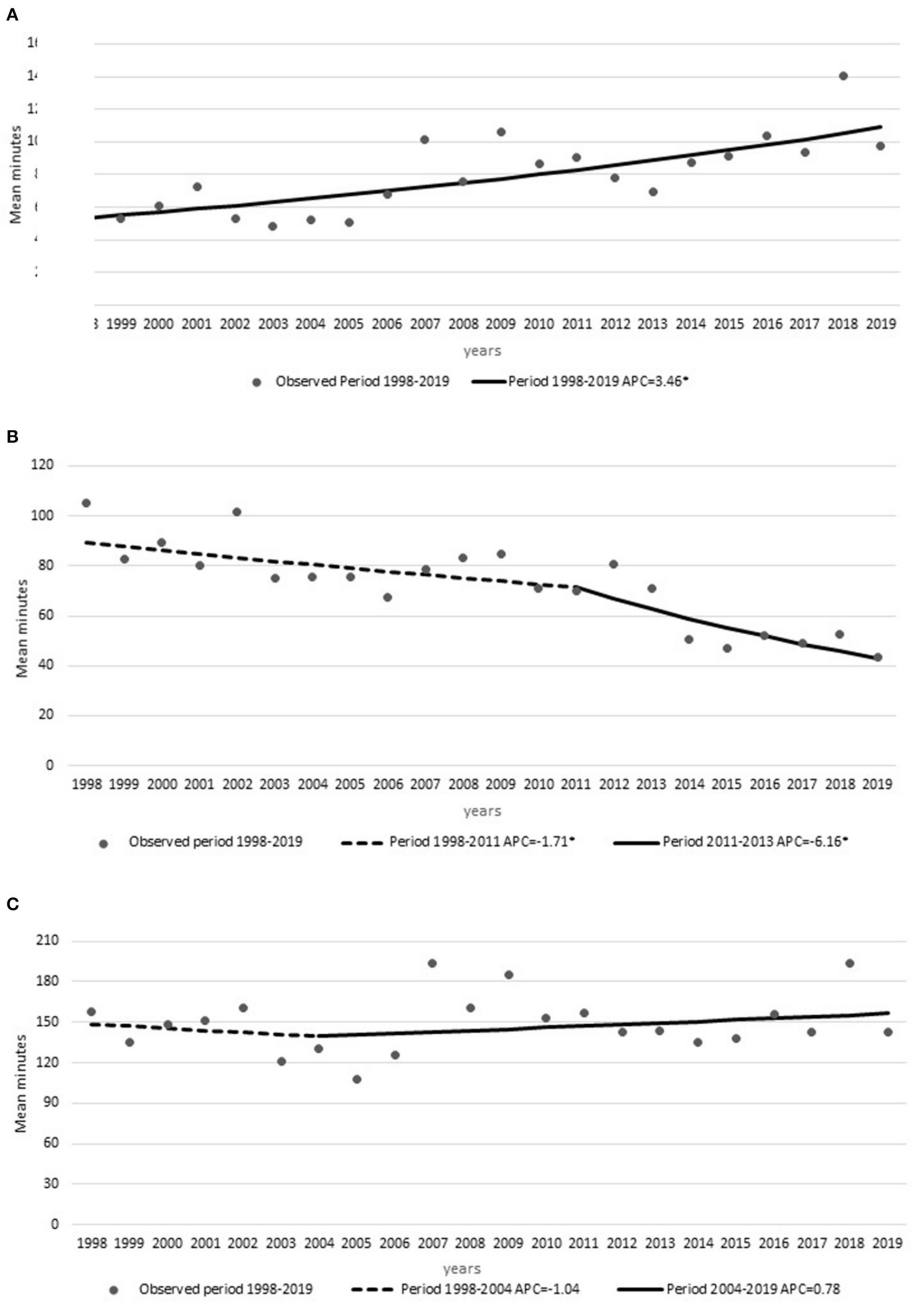
Time metrics: annual percent changes in **(A)** symptom onset-to-door times, **(B)** door-to-needle times, and **(C)** symptom onset-to-needle times from 1998 to 2019 with Joinpoints. *Indicates were the annual percent change (APC) is significantly different from zero at the alpha = 0.05 level.

The time metrics showed an increase in OTD times (APC, +3.45 min) with no Joinpoints and a decrease in DTN times (AAPC, −4.1 min; 95% CI, −5.1 to −3) with one Joinpoint in 2011. In this case, the APCs were −1.71 min from 1998 to 2011 and −6.16 min from 2011 to 2019 (Figures 2A,B). The OTN times showed a Joinpoint in 2010, with an initial increase (APC, +0.42 min) from 1998 to 2010 and then a significant decrease (APC, −2.49 min) from 2010 to 2019 (Figure 2C). The significant change in DTN times from 2011 onward concurred with several changes in workflow, particularly the IVT written consent was eliminated, laboratory results were no longer waited for, and r-TPA was started in the CT suite (Supplementary Table 2).

**Figure 3:**
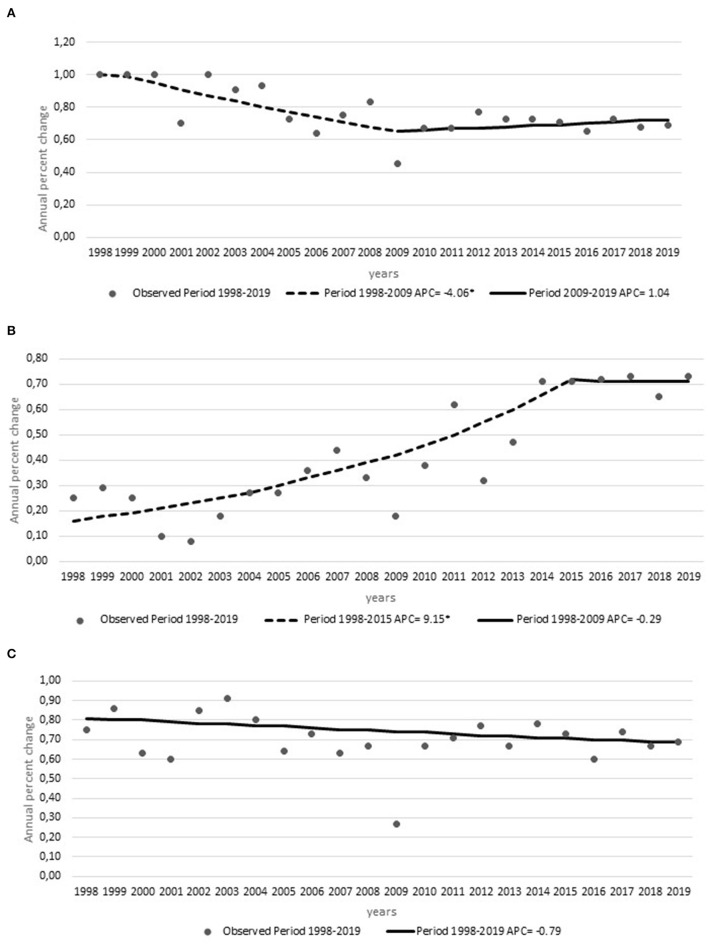
Compliance metrics: annual percent changes in the **(A)** frequency of thrombolysis within 120 min of symptom onset, **(B)** frequency of door-to-needle time within 60 min, and **(C)** frequency of thrombolysis within 180 min of symptom onset from 1998 to 2019 with Joinpoints. *Indicates were the annual percent change (APC) is significantly different from zero at the alpha = 0.05 level.

The compliance with performance measures showed a decrease in the proportion of patients arriving within 120 min of symptom onset with one Joinpoint in 2009 (APC, −4.06%), where the trend changed to a slight increase (APC, +1.04%). This can be associated with the stroke code implementation in 2008 (Supplementary Table 2). The regression also showed an increase in the frequency of patients treated with thrombolysis within 60 min of arrival to the emergency department (APC, +9.15) with one Joinpoint in 2015 when it stabilized (APC, −0.29%) (Figures 3A,B). The proportion of patients treated within 180 min from symptom onset was stable (APC, 0.79%) without a Joinpoint (Figure 3C).

The frequencies of missing data were as follows: IVT, 0%; OTD, 5%; DTN, 5.6%; OTN, 7.2%.

## Discussion

Our results show that the frequency of IVT persistently increased by an average percent change of 7% per year, which is consistent with a continuous process of improvement. In the past decade, several other studies have shown similarly continuously increasing rates in different countries throughout the world (20–24).

The significant change in trend in thrombolysis utilization from 2007 onward was associated with two relevant interventions and one policy change. The behavioral impact of these factors can be described with the BCW conceptual framework. The extension of the time window was a specific opportunity (O), and the organization of the stroke code boosted the team capabilities (C). Institutional quality parameters were periodically published on the website and meant recognition of relevance with motivational (M) impact for the team. Furthermore, the organization of the stroke code was a system change with a multilevel, multicomponent intervention that included at least the five interventions associated with improving the rates of IVT (i.e., education, training, persuasion, environment restructuring, and enablement) (12).

The significant change in DTN times from 2011 onward was concurrent with several changes in workflow implemented to lower in-hospital barriers. These interventions have resulted in reduced DTN times in several studies (7, 12, 14, 25).

The increase in OTD times could be associated with higher confidence of the team in selecting late comers and increasing time windows. The small but significant improvement in the trend of the proportion of patients arriving within 120 min of symptom in 2009 can be associated with the implementation in 2008 of institutional EMS training, prenotification, and annual stroke awareness campaigns that have been previously shown to be significant interventions (14, 15). The continuous increase in the proportion of patients being treated with thrombolysis within 60 min of emergency department arrival is most probably due to the sum of interventions throughout the years and a learning curve of the treating teams. Interventions, such as setting another DTN performance measure of 50% being treated within 45 min, are being implemented with success in several centers and could further decrease this metric (26). Other effective improvement strategies are the transport of patients by EMS directly to the scanner and rapid registration of patients (25, 27). Furthermore, there is cumulating evidence that pre-hospital interventions aimed to optimize workflow contributed to reducing pre-hospital and in-hospital delays (28, 29).

In this analysis, we did not focus on patients' characteristics or factors determining thrombolysis use that have also been shown to significantly delay DTN times (30). We analyzed these factors in another recent publication showing that unrecognized stroke by the triage system, vertebrobasilar territory strokes, hypertension control, severe strokes needing intubation, and possible contraindications (oral anticoagulation) was significantly associated with DTN times over 60 min (31).

This observational study has several limitations and potential biases. We only included interventions that are directly associated with IVT and excluded other hospital-wide quality improvements, such as the National and Joint Commission International accreditation processes, emergency department and EMS organizational and staffing modifications, and changes in the imaging department, such as CT availability. It is the experience of a single academic medical center serving an urban population. We do not have data on the transport system used during the study period.

Strengths of this study include its long period of observation, low frequency of missing data, use of a novel trend regression analysis, and analysis of interventions based on the BCW framework.

Several studies that have described increasing thrombolysis rates associated with different interventions have analyzed mostly their effectiveness (22, 32, 33). Our analysis focused on the significant change in trends and the interventions associated with those changes. IVT in AIS is a multistep clinical decision process, involving many disciplines and components of the healthcare systems and with a short time window (34). Thus, it requires complex interventions, difficult to implement, maintain in time, and reproduce (13). We chose to characterize the interventions using the BCW framework, which is based on the COM-B behavior system as it seems to be robust in improving the design and implementation of evidence-based complex interventions (12).

Our findings suggest that those interventions that fulfill strategic components of the BCW conceptual framework have a greater impact in changing the trends in thrombolysis utilizations rates. A close observation of the core components of opportunity, capability, and motivation may be a practical guide to promote changes in behavior in complex clinical processes. Our results also suggest that the benefit of in-hospital interventions has a ceiling effect and that further improvement is the realm of pre-hospital interventions reducing both pre-hospital and in-hospital delays, increasing the number of patients having timely access to IVT (35).

## Data Availability Statement

The raw data supporting the conclusions of this article will be made available by the authors, without undue reservation.

## Ethics Statement

The studies involving human participants were reviewed and approved by Comité ético científico, facultad de medicina Clínica Alemana, Universidad del Desarrollo. The patients/participants provided their written informed consent to participate in this study.

## Author Contributions

VO, LH, and PL conceived the idea and were responsible for the literature search and planning of the analysis. VO and PL were responsible for the data base organization and wrote the first draft. VO, PL, LH, and CV were responsible for database administration and analysis of data. VO, LH, PL, AB, and AH participated in the interpretation and critical review of the intellectual content of the study. All authors approved the final manuscript.

## Funding

This study received funding from Clínica Alemana de Santiago, Boehringer Ingelheim and Lundbeck Chile. The funders were not involved in the study design, collection, analysis, interpretation of data, and the writing of this article or the decision to submit it for publication.

## Conflict of Interest

VO reports receiving research grants from Clínica Alemana de Santiago, Boehringer-Ingelheim, and ANID. PL reports research support from Clínica Alemana de Santiago and Boehringer Ingelheim, research grants from The George Institute and Clínica Alemana de Santiago during the conduct of the study, unrestricted research grants from Lundbeck and Boehringer Ingelheim, personal fees from AstraZeneca and Bayer as SOCRATES and ESUS NAVIGATE trials national leader, and a Chilean Government research grant for the ÑANDU project outside the submitted work. AB received a research grant from Clínica Alemana de Santiago for the RECCA registry. The remaining authors declare that the research was conducted in the absence of any commercial or financial relationships that could be construed as a potential conflict of interest.

## Publisher's Note

All claims expressed in this article are solely those of the authors and do not necessarily represent those of their affiliated organizations, or those of the publisher, the editors and the reviewers. Any product that may be evaluated in this article, or claim that may be made by its manufacturer, is not guaranteed or endorsed by the publisher.

## References

[1] Emberson J Lees KR Lyden P Blackwell L Albers G Bluhmki E. Effect of treatment delay, age, and stroke severity on the effects of intravenous thrombolysis with alteplase for acute ischaemic stroke: a meta-analysis of individual patient data from randomised trials. Lancet Lond Engl. (2014) 384:1929–35. 10.1016/S0140-6736(14)60584-5 25106063 PMC4441266

[2] Qin B Zhao M-J Chen H Qin H Zhao L Fu L. Real-world outcomes of acute ischemic stroke treatment with intravenous thrombolysis: a systematic review and meta-analysis. J Stroke Cerebrovasc Dis Off J Natl Stroke Assoc. (2018) 27:3542–8. 10.1016/j.jstrokecerebrovasdis.2018.08.015 30201455

[3] Hoffmeister L Lavados PM Mar J Comas M Arrospide A Castells X. Minimum intravenous thrombolysis utilization rates in acute ischemic stroke to achieve population effects on disability: a discrete-event simulation model. J Neurol Sci. (2016) 365:59–64. 10.1016/j.jns.2016.04.005 27206876

[4] Zhou Y Yan S Song X Gong Y Li W Wang M. Intravenous thrombolytic therapy for acute ischemic stroke in Hubei, China: a survey of thrombolysis rate and barriers. BMC Neurol. (2019) 19:202. 10.1186/s12883-019-1418-z 31438899 PMC6704516

[5] Alonso de. Leciñana María, Mazya Michael V, Kostulas Nikolaos, Del Brutto Oscar H, Abanto Carlos, et al. Stroke care and application of thrombolysis in ibero-America. Stroke. (2019) 50:2507–12. 10.1161/STROKEAHA.119.025668 31670921

[6] Aguiar de. Sousa D, von Martial R, Abilleira S, Gattringer T, Kobayashi A, et al. Access to and delivery of acute ischaemic stroke treatments: a survey of national scientific societies and stroke experts in 44 European countries. Eur Stroke J. (2019) 4:13–28. 10.1177/2396987318786023 31165091 PMC6533860

[7] Eissa A Krass I Bajorek BV. Barriers to the utilization of thrombolysis for acute ischaemic stroke. J Clin Pharm Ther. (2012) 37:399–409. 10.1111/j.1365-2710.2011.01329.x 22384796

[8] Messé SR Khatri P Reeves MJ Smith EE Saver JL Bhatt DL. Why are acute ischemic stroke patients not receiving IV tPA? Results from a national registry. Neurology. (2016) 87:1565–74. 10.1212/WNL.0000000000003198 27629092 PMC5067546

[9] De Brún A Flynn D Ternent L Price CI Rodgers H Ford GA. Factors that influence clinicians' decisions to offer intravenous alteplase in acute ischemic stroke patients with uncertain treatment indication: Results of a discrete choice experiment. Int J Stroke Off J Int Stroke Soc. (2018) 13:74–82. 10.1177/1747493017690755 28134031

[10] Ormseth CH Sheth KN Saver JL Fonarow GC Schwamm LH. The American heart association's get with the guidelines (GWTG)-stroke development and impact on stroke care. Stroke Vasc Neurol. (2017) 2:94–105. 10.1136/svn-2017-000092 28959497 PMC5600018

[11] Paul CL Ryan A Rose S Attia JR Kerr E Koller C. How can we improve stroke thrombolysis rates? A review of health system factors and approaches associated with thrombolysis administration rates in acute stroke care. Implement Sci IS. (2016) 11:51. 10.1186/s13012-016-0414-6 27059183 PMC4825073

[12] Michie S van Stralen MM West R. The behaviour change wheel: A new method for characterising and designing behaviour change interventions. Implement Sci. (2011) 6:42. 10.1186/1748-5908-6-42 21513547 PMC3096582

[13] Hasnain MG Attia JR Akter S Rahman T Hall A Hubbard IJ. Effectiveness of interventions to improve rates of intravenous thrombolysis using behaviour change wheel functions: a systematic review and meta-analysis. Implement Sci IS. (2020) 15:98. 10.1186/s13012-020-01054-3 33148294 PMC7641813

[14] Huang Q Zhang J-Z Xu W-D Wu J. Generalization of the right acute stroke promotive strategies in reducing delays of intravenous thrombolysis for acute ischemic stroke: a meta-analysis. Medicine. (2018) 97:e11205. 10.1097/MD.0000000000011205 29924046 PMC6024468

[15] McDermott M Skolarus LE Burke JF A A systematic review and meta-analysis of interventions to increase stroke thrombolysis. BMC Neurol. (2019) 19:86. 10.1186/s12883-019-1298-2 31053101 PMC6500041

[16] Sacco RL Kasner SE Broderick JP Caplan LR Connors JJB Culebras A. An updated definition of stroke for the 21st century: a statement for healthcare professionals from the American heart association/American stroke association. Stroke. (2013) 44:2064–89. 10.1161/STR.0b013e318296aeca 23652265 PMC11078537

[17] National Institute of Neurological Disorders and Stroke rt-PA Stroke Study Group. Tissue plasminogen activator for acute ischemic stroke. N Engl J Med. (1995) 333:1581–8. 10.1056/NEJM199512143332401 7477192

[18] Powers WJ Rabinstein AA Ackerson T Adeoye OM Bambakidis NC Becker K. Guidelines for the early management of patients with acute ischemic stroke: 2019 update to the 2018 guidelines for the early management of acute ischemic stroke: a guideline for healthcare professionals from the American heart association/American stroke association. Stroke. (2019) 50:e344–418. 10.1161/STR.0000000000000211 31662037

[19] Joinpoint Regression Program. Available online at: https://surveillance.cancer.gov/joinpoint/ (accessed October 6, 2021).

[20] Nagaraja N Kubilis PS Hoh BL Wilson CA Khanna AY Kelly AG. Trends of Acute ischemic stroke reperfusion therapies from 2012 to 2016 in the United States. World Neurosurg. (2021) 150:e621–30. 10.1016/j.wneu.2021.03.073 33757890

[21] Moro CHC Gonçalves ARR Longo AL Fonseca PG Harger R Gomes DB. Trends of the incidence of ischemic stroke thrombolysis over seven years and one-year outcome: a population-based study in Joinville, Brazil. Cerebrovasc Dis Extra. (2013) 3:156–66. 10.1159/000356984 24570681 PMC3924708

[22] Scherf S Limburg M Wimmers R Middelkoop I Lingsma H. Increase in national intravenous thrombolysis rates for ischaemic stroke between 2005 and 2012: is bigger better? BMC Neurol. (2016) 16:53. 10.1186/s12883-016-0574-7 27103535 PMC4839134

[23] Singer OC Hamann GF Misselwitz B Steinmetz H Foerch C Arbeitsgruppe Schlaganfall Hessen. Time trends in systemic thrombolysis in a large hospital-based stroke registry. Cerebrovasc Dis Basel Switz. (2012) 33:316–21. 10.1159/000335816 22343969

[24] Asaithambi G Tong X Lakshminarayan K Coleman King SM George MG. Current trends in the acute treatment of ischemic stroke: analysis from the Paul Coverdell national acute stroke program. J Neurointerventional Surg. (2020) 12:574–8. 10.1136/neurintsurg-2019-015133 31653755 PMC7558219

[25] Kamal N Holodinsky JK Stephenson C Kashayp D Demchuk AM Hill MD. Improving door-to-needle times for acute ischemic stroke: effect of rapid patient registration, moving directly to computed tomography, and giving alteplase at the computed tomography scanner. Circ Cardiovasc Qual Outcomes. (2017) 10: 10.1161/CIRCOUTCOMES.116.003242 28096208

[26] Kamal N Smith EE Jeerakathil T Hill MD. Thrombolysis: improving door-to-needle times for ischemic stroke treatment - a narrative review. Int J Stroke Off J Int Stroke Soc. (2018) 13:268–76. 10.1177/1747493017743060 29140185

[27] Zinkstok SM Beenen LF Luitse JS Majoie CB Nederkoorn PJ Roos YB. Thrombolysis in stroke within 30 minutes: results of the acute brain care intervention study. PLoS ONE. (2016) 11:e0166668. 10.1371/journal.pone.0166668 27861540 PMC5115772

[28] Schott AM Termoz A Viprey M Tazarourte K Vecchia CD Bravant E. Short and long-term impact of four sets of actions on acute ischemic stroke management in Rhône County, a population based before-and-after prospective study. BMC Health Serv Res. (2021) 21:12. 10.1186/s12913-020-05982-0 33397363 PMC7783982

[29] Grotta JC Yamal J-M Parker SA Rajan SS Gonzales NR Jones WJ. Prospective, multicenter, controlled trial of mobile stroke units. N Engl J Med. (2021) 385:971–81. 10.1056/NEJMoa2103879 34496173

[30] Kamal N Sheng S Xian Y Matsouaka R Hill MD Bhatt DL. Delays in door-to-needle times and their impact on treatment time and outcomes in get with the guidelines-stroke. Stroke. (2017) 48:946–54. 10.1161/STROKEAHA.116.015712 28228574

[31] Brunser AM Mazzon E Muñoz P Hoppe A Lavados PM Rojo A. [Determinants of door to needle time for intravenous thrombolysis in acute ischemic stroke]. Rev Med Chil. (2020) 148:1090–5. 10.4067/S0034-98872020000801090 33399775

[32] Schwamm LH Ali SF Reeves MJ Smith EE Saver JL Messe S. Temporal trends in patient characteristics and treatment with intravenous thrombolysis among acute ischemic stroke patients at Get With The Guidelines-Stroke hospitals. Circ Cardiovasc Qual Outcomes. (2013) 6:543–9. 10.1161/CIRCOUTCOMES.111.000095 24046398

[33] Campbell BCV Khatri P. Stroke. Lancet Lond Engl. (2020) 396:129–42. 10.1016/S0140-6736(20)31179-X 32653056

[34] Paul CL Levi CR D'Este CA Parsons MW Bladin CF Lindley RI. Thrombolysis ImPlementation in Stroke (TIPS): evaluating the effectiveness of a strategy to increase the adoption of best evidence practice – protocol for a cluster randomised controlled trial in acute stroke care. Implement Sci IS. (2014) 9:38. 10.1186/1748-5908-9-38 24666591 PMC4016636

[35] Chowdhury SZ Baskar PS Bhaskar S. Effect of prehospital workflow optimization on treatment delays and clinical outcomes in acute ischemic stroke: a systematic review and meta-analysis. Acad Emerg Med Off J Soc Acad Emerg Med. (2021) 28:781–801. 10.1111/acem.14204 33387368

