# Supplementary material for: Temporal Trends of Intravenous Thrombolysis Utilization in Acute Ischemic Stroke in a Prospective Cohort From 1998 to 2019: Modeling Based on Joinpoint Regression
Source: Front Neurol. 2022 Apr 8;13:851498. doi: 10.3389/fneur.2022.851498 (PMC9028765; doi:10.3389/fneur.2022.851498)
Supplement: Supplementary file 1 [file Table_1.DOCX]

Supplementary material 1

Table 1. Organizational and protocol modifications classified according to the behavioral change wheel conceptual framework intervention components and policy category

| **Organizational or protocol modifications** | **Year** | **Intervention components according to the Behavioral Change Wheel conceptual framework** | | | | | | | | | **Policy category** |
| --- | --- | --- | --- | --- | --- | --- | --- | --- | --- | --- | --- |
|  |  | Education | Persuasion | Incentivization | Coercion | Training | Restriction | Environment  Restructuring | Modeling | Enablement | Guidelines and protocols |
| First thrombolysis protocol, neuroimaging protocol included NCCT*, cerebral CTA†, DWI-MRI‡ | 1997 September | x | x |  |  | x | x | x |  | x | x |
| Prospective stroke registry | 1997 September | x | x |  |  | x |  |  |  |  |  |
| Vascular neurology fellowship. | 2001 | x | x | x |  | x |  |  |  |  |  |
| Vascular neurology rounds. | 2001 | x | x |  |  | x |  |  |  |  |  |
| Mobile stroke unit. | 2005 | x | x | x |  | x |  |  | x | x |  |
| Cervical artery CTA† or MRA‡ in all cases | 2005 | x |  |  |  | x |  |  |  | x | x |
| Regular training of nurses and paramedics | 2005 | x | x |  |  | x |  |  |  |  |  |
| Stroke code organization. | 2008 | x | x |  |  | x | x | x | x | x | x |
| DTN§<60 minutes as a performance measure | 2008 | x | x |  | x | x |  | x | x | x | x |
| Extension of the therapeutic window to 4,5 hours. | 2008 | x |  |  |  | x | x |  |  | x | x |
| Elimination of the need for written informed consent. | 2011 | x |  |  |  | x | x |  | x |  | x |
| Elimination of the need to wait for the laboratory results. | 2011 | x |  |  |  | x |  |  | x |  | x |
| Bolus delivered immediately after NCCT* | 2012 | x |  |  |  | x |  | x | x |  | x |
| No exclusion for NIHSS\|\| < 5 | 2013 | x |  |  |  | x | x |  |  | x | x |
| No warning for NIHSS > 22 | 2013 | x |  |  |  | x | x |  |  | x | x |
| Flair MRI¶ for wake-up strokes | 2015 | x |  |  |  | x |  | x | x |  | x |
| CT perfusion for wake-up strokes, unknown SOT or >6 hrs. from OTD** | 2018 | x | x |  |  | x |  | x | x | x | x |

* NCCT: Non-contrast computer tomography. †CTA: Computed tomography angiography. ‡DWI-MRI Diffusion-weighted magnetic resonance imaging. ‡MRA: magnetic resonance angiography. §DNT: Door-to-needle times ||NIHSS: National institutes of health stroke scale. ¶MRI: magnetic resonance imaging. **OTD: Symptom onset-to-door times.
