# Supplementary material for: Temporal Trends of Intravenous Thrombolysis Utilization in Acute Ischemic Stroke in a Prospective Cohort From 1998 to 2019: Modeling Based on Joinpoint Regression
Source: Front Neurol. 2022 Apr 8;13:851498. doi: 10.3389/fneur.2022.851498 (PMC9028765; doi:10.3389/fneur.2022.851498)
Supplement: Supplementary file 2 [file Table_2.DOCX]

Supplementary material 2

Table 2. Description of interventions or protocol modifications.

| **Organizational or protocol modifications** | **Intervention description** |
| --- | --- |
| First thrombolysis protocol | Thrombolysis protocol based on NINDS* trial, with all the exclusion criteria and warnings. This included production and dissemination of documents, teaching about, and promoting the concept that ‘time is brain’ and thrombolysis benefits, training in clinical and neuroimaging protocol and NIHSS†, regular feedback, and availability of Alteplase and labetalol. Laboratory and neuroimaging examinations were standardized according to the protocol. |
| Prospective stroke registry | Prospective stroke registry of all patients with stroke admitted to the institution. This included design, education about the need to have prospective data, and training in data entry and communication. |
| Vascular neurology fellowship | Organization of a 1-year vascular neurology fellowship, who participates in most of recanalization reperfusion procedures, together with the neurologist on call at the emergency department. This included education and training in vascular neurology and patient care, institutional protocols, and data entry to the registry. |
| Vascular neurology rounds | Organization of the daily vascular neurology rounds, where all admitted patients with stroke are evaluated by a vascular neurologist and vascular neurology fellow. Patient care was reviewed according to existing protocols and evidence. Education and training were provided on a patient-to-patient basis. |
| Mobile stroke unit | Organization of a mobile stroke unit, which included a speech therapist, a physiotherapist, occupational therapists, a vascular neurology fellow, and the vascular neurologist on call. This included regular education in vascular neurology diagnosis, treatment, prevention, and rehabilitation concepts. Daily discussions about patient care, goals, and discharge plans were organized. |
| Cervical artery CTA‡ or MRA§ in all cases | A vascular study of the neck was included in all cases that had brain CTA or MRA. This included education and training activities. |
| Regular training of emergency staff | Education in stroke recognition, care, and protocols. This included regular weekly training sessions with different shifts. Communication was encouraged, and feedback was provided. |
| Stroke code organization | Organization of the stroke code in the pre-hospital and emergency department. All patients suspected stroke with a Cincinnati scale score above 1 and <6 h from symptom onset were coded. Anyone could code a patient. Ready standard laboratory orders and imaging protocol were organized, and the neurologist on call was immediately made aware. The implementation included the preparation of a thrombolysis toolbox always ready to be managed by emergency nurses. Education and training were provided on a hospital-wide basis including dissemination of the results of its implementation in centers. These patients were prioritized over others. |
| DTN§ > 60 minutes as a performance measure | Definition of the door-to-needle time over 60 minutes as a performance measure in our institution, which is made public on the web. All cases surpassing this time were evaluated in a weekly vascular neurology meeting. This was part of the stroke code organization and as an endpoint was included in all education, training, and communication opportunities. |
| Extension of the therapeutic window to 4.5 hours | Extension of the therapeutic window from 3 to 4.5 hours, with no exclusions regarding age or previous diabetes. |
| Elimination of the need for written informed consent | Elimination of the need for written informed consent from the patient or family/next of kin to treat with thrombolysis. Patients can opt out of thrombolysis. This protocol change was part of the updates performed because of the participation of the center in the ENCHANTED clinical trial. It included education, training, and modeling based on other acute care protocols such as meningitis, status epilepticus, acute myocardial infarction, and pulmonary embolism that did not need consent. |
| Elimination of the need to wait for the laboratory results | The elimination of the need to have the results of the laboratory examinations before bolus in non-anticoagulated patients. This protocol change was part of the updates performed because of the participation of the center in the ENCHANTED clinical trial. It included education, training, and modeling based on other acute care protocols such as meningitis, which started treatment before laboratory results. |
| Bolus delivered immediately after NCCT | A change in the thrombolysis protocol allowing the bolus to be delivered immediately after NCCT in the CT\|\| room. A renovation of the neuroimaging department including CT rooms facilitated this process. This protocol change was part of the updates performed because of the participation of the center in the ENCHANTED clinical trial. It included education, training, and modeling based on the published experience of other centers. |
| No exclusion for NIHSS scores of <5 | The elimination of the exclusion of patients with NIHSS scores of <5 or isolated symptoms. The decision was left to the vascular neurologist on call. All patients with a demonstrable occluded artery were treated with thrombolysis according to the protocol independently of symptoms. This included education and training. |
| No warning for NIHSS scores of >22 | The cautionary warning in patients with NIHSS scores of ≥22 was eliminated especially in patients programed for thrombectomy. This included education and training. |
|  |  |
| FLAIR MRI¶ for wake-up strokes | The update of the imaging protocol adding FLAIR MRI for wake-up strokes with low NIHSS scores. It included education, training, and modeling based on the published experience of other centers. |
| CT perfusion for wake-up strokes, unknown SOT or >6 horas from OTD** | The update of the imaging protocol adding CT perfusion for wake-up strokes, unknown onset time, or patients arriving beyond time windows, using automated software. It included education, training, and modeling based on the published experience of other centers. |

*NINDS: National Institute of Neurological Disorders and Stroke. †NIHSS: National Institutes of Health Stroke Scale. ‡CTA: Computed tomography angiography. §MRA: Magnetic resonance angiography. §DNT: door-to-needle times. NCCT: non-contrast computed tomography scan. ||CT: computed tomography. ¶MRI: magnetic resonance imaging. **OTD: Symptom onset-to-door times.
